# Housing First Reduces Re-offending among Formerly Homeless Adults with Mental Disorders: Results of a Randomized Controlled Trial

**DOI:** 10.1371/journal.pone.0072946

**Published:** 2013-09-04

**Authors:** Julian M. Somers, Stefanie N. Rezansoff, Akm Moniruzzaman, Anita Palepu, Michelle Patterson

**Affiliations:** 1 Faculty of Health Sciences, Simon Fraser University, Burnaby, British Columbia, Canada; 2 Faculty of Medicine, University of British Columbia, Vancouver, British Columbia, Canada; University of Western Brittany, France

## Abstract

**Background:**

Homelessness and mental illness have a strong association with public disorder and criminality. Experimental evidence indicates that Housing First (HF) increases housing stability and perceived choice among those experiencing chronic homelessness and mental disorders. HF is also associated with lower residential costs than common alternative approaches. Few studies have examined the effect of HF on criminal behavior.

**Methods:**

Individuals meeting criteria for homelessness and a current mental disorder were randomized to one of three conditions treatment as usual (reference); scattered site HF; and congregate HF. Administrative data concerning justice system events were linked in order to study prior histories of offending and to test the relationship between housing status and offending following randomization for up to two years.

**Results:**

The majority of the sample (67%) was involved with the justice system, with a mean of 8.07 convictions per person in the ten years prior to recruitment. The most common category of crime was “property offences” (mean = 4.09). Following randomization, the scattered site HF condition was associated with significantly lower numbers of sentences than treatment as usual (Adjusted IRR = 0.29; 95% CI 0.12–0.72). Congregate HF was associated with a marginally significant reduction in sentences compared to treatment as usual (Adjusted IRR = 0.55; 95% CI: 0.26–1.14).

**Conclusions:**

This study is the first randomized controlled trial to demonstrate benefits of HF among a homeless sample with mental illness in the domain of public safety and crime. Our sample was frequently involved with the justice system, with great personal and societal costs. Further implementation of HF is strongly indicated, particularly in the scattered site format. Research examining interdependencies between housing, health, and the justice system is indicated.

**Trial registration:**

ISRCTN57595077

## Introduction

People who are both homeless and mentally ill are at very high risk of being arrested and involved with the criminal justice system [1], [2]. Considerable public costs have been associated with service use among this subgroup, with justice system involvement accounting for a significant proportion of these expenditures [3]. Healthcare and housing interventions have been shown to produce multiple benefits among the homeless mentally ill, particularly the model known as Housing First (HF) [4]. HF is characterized by rapid rehousing in permanent, market accommodations without requirements around sobriety or treatment adherence, and facilitating access to specific resources (e.g., health, social, vocational) to support the attainment of client centered goals [5], [6]. Few studies have examined the effectiveness of HF on justice system outcomes, and none have used an experimental design.

Worldwide, people with mental disorders are significantly over-represented in prison populations [7]. An estimated one million persons with mental disorders are involved with the US justice system alone [8]. The intersection between mental health and criminal justice is both striking and yet poorly understood, and has been described as a “new frontier” for health services research [9].

Interestingly, little evidence exists to support a direct causal link between mental illness and offending. Instead, indirect pathways appear to account for the association between mental disorders and crime [8] including the experience of poverty, social marginalization, unemployment, and exposure to substance use, crime and victimization [10]. The treatment of mental disorders among offenders is necessary for the promotion of health [11]. However, the goal of reducing offending among people with mental illness requires attention to a broad range of factors associated with recidivism [12]. Chronic homelessness is a powerful mediator of crime [13], [14] and is disproportionately experienced by persons with mental disorders [15]. Homelessness can be both a cause and a consequence of involvement with the justice system. Individuals commit more offences after becoming homeless than before [16] and, in a reciprocal manner, incarceration contributes to homelessness through the destabilization of housing, unemployment, and the erosion of human rights [17]. The rate of recent homelessness among jail inmates is reportedly 7.5 to 11.3 times higher than in the general population after adjusting for age, ethnicity and gender [18].

Very few interventions for persons who are both homeless and mentally ill have been evaluated for their effects on crime or public safety. Calsyn and colleagues [19] report results of a randomized controlled trial involving participants who were homeless and who had both a serious mental disorder and a substance use disorder. Over two years of follow up they found no differences in criminal justice outcomes between participants receiving Assertive Community Treatment (ACT), integrated “dual diagnosis” treatment (IT), or usual care. The authors caution that “although ACT and IT programs have produced better outcomes than other management approaches in terms of hospitalization, housing, mental health and sometimes substance abuse, clinicians should not expect that these positive outcomes will necessarily extend to criminal behavior” ([19], p.245).

A small number of more recent studies using the HF approach have reported significant and positive impacts on crime and public safety. These studies have utilized the traditional scattered site model in which participants are dispersed in market accommodations [20] as well as congregate or “project based” configurations where participants are supported together in a single building [21], [22]. The overwhelming majority of offences committed by participants in these studies were property-type crimes. Results illustrate the promise of HF to reduce offending among chronically homeless individuals with severe alcohol problems [21], [22], and also among homeless individuals with work-related disabilities [20]. These non-experimental findings suggest that HF may mediate improvements in public safety among formerly homeless individuals with varying needs. However, it is less well known whether HF promotes reductions in crime among people with mental disorders, with or without concurrent substance dependence. This omission from the research literature is important because HF was developed and designed to promote recovery among those who are homeless and mentally ill [23].

The present study uses a randomized controlled trial design to study the effectiveness of HF as a means of reducing crime among individuals who meet criteria for current homelessness and the presence of a mental disorder, and who had previous involvement with the justice system. We use a three arm design, with randomization to scattered site HF (SS), congregate HF (CONG), or treatment as usual (TAU). Our primary hypothesis is that HF, whether configured in SS or CONG format, is associated with significantly lower re-offence rates than TAU.

## Methods

### Participants

**Figure 1:**
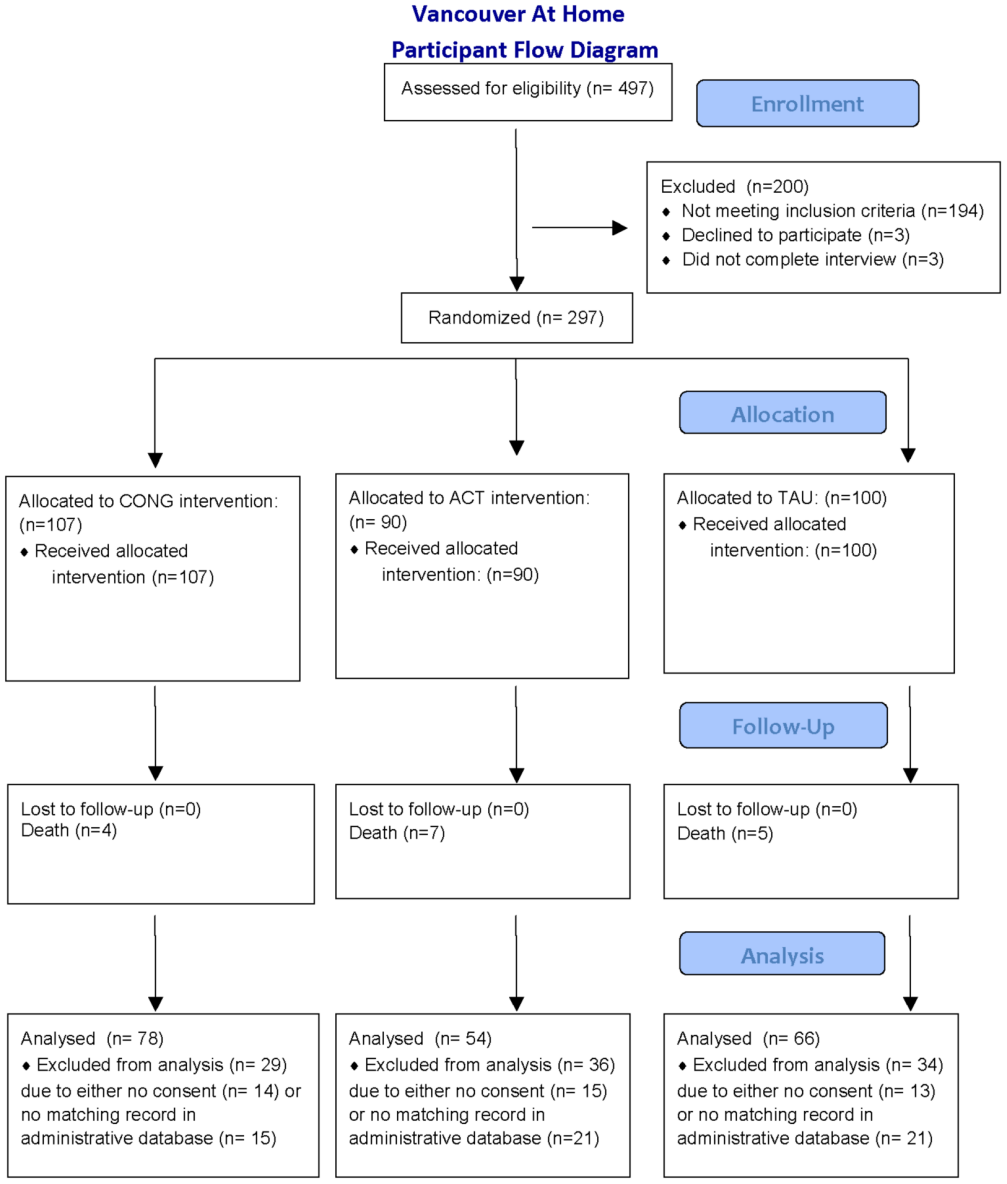
Participant flow diagram.

The study underwent institutional review and was approved by the Research Ethics Boards at Simon Fraser University and the University of British Columbia. Participants (n = 297) comprise the cohort enrolled in the Vancouver at home study: HF plus ACT versus congregate housing plus supports versus TAU (ISRCTN57595077; http://www.controlled-trials.com/ISRCTN57595077). The study is a three-arm, randomized controlled trial involving adults who are homeless and have a mental disorder (see Figure 1). Eligibility criteria included legal adult status (19 years of age or over), presence of a current mental disorder on the MINI International Neuropsychiatric Interview (MINI) [24] and being absolutely homeless or precariously housed. Mental disorder status was confirmed through written diagnosis from physicians or other service providers wherever possible. “Absolute homelessness” was defined as living on the streets or in a shelter for at least the past seven nights with little chance of obtaining secure accommodation. “Precariously housed” was defined as living in a rooming house, hotel or other form of transitional housing with at least two episodes of absolute homelessness in the past year. Additional inclusion criteria were: hospitalization in the past two years for a psychiatric reason; justice system involvement in the past two years; and current low level of community functioning as indicated by Multnomah Community Ability Scale (MCAS [25]) score not higher than 62.

Participants were recruited through referral from over 40 agencies representing approximately 13 different types of services available to homeless adults in Vancouver. The majority of participants were recruited from: homeless shelters; drop-in centers; homeless outreach teams; hospitals; community mental health teams and criminal justice programs. Computerized randomization was performed in the field using a wireless link to a central data centre. An adaptive randomization procedure was used, meaning that the probability of assignment to groups changed as a function of the number of participants previously assigned. Additional study details, including interviews and measures not included in the current study, are published separately [26], [27].

Individuals were invited to complete an eligibility screener and informed consent. Development of the informed consent protocol was preceded by field-testing including cognitive interviewing to ensure that materials were presented understandably [28]. All interviews were conducted by experienced research staff members who received ongoing support and supervision. Interviews were discontinued if participants’ mental status was compromised by acute symptoms or substance use. If eligible and willing to participate, participants completed a series of detailed interviewer-administered baseline questionnaires addressing: socio-demographic characteristics, symptoms of mental illness, substance use, and physical health. All participants received a cash honorarium for the screening questionnaire ($5.00) and the baseline interview ($25.00). The informed consent procedure included separate consideration (and specific consent) for the receipt of administrative records including encounters with the justice system. For consenting individuals, these data were then obtained from the relevant department (Provincial Ministry of Justice).

### Interventions

Two HF interventions (scattered-site housing with ACT and congregate housing with on-site supports) were compared to TAU. Both HF interventions underwent fidelity assessments guided by the HF fidelity scale developed by Pathways to Housing [29]. The Pathways to Housing program in New York is generally credited with the development of HF [30], [31], although other variants have emerged.

In the Scattered Site (SS) condition, individuals were provided with a choice of housing (typically from among 2–3 available units) in buildings where at most 20% of the units were occupied by study participants and in locations scattered throughout the city of Vancouver. An inventory of units was developed and maintained by a dedicated housing portfolio manager. An Assertive Community Treatment (ACT) team was created to support up to 100 participants recruited through the study. The ACT team served only those individuals randomized to the SS condition, and was modeled on the Pathways program [23]. Services provided by the ACT team included psychiatry and primary healthcare, as well as social and vocational rehabilitation. Services were available 24 hours a day, seven days a week, and were typically provided in participants’ homes. Participants were required to meet with a member of the program team once per week, but there were no other requirements regarding compliance with treatment. Participant choice was emphasized and a harm reduction approach to substance use was promoted.

The Congregate (CONG) arm of the study was implemented in a former hotel, in which all residents were randomized via the study protocol. Participants in CONG were provided with on-site supports that were intended to match the overall intensity and composition of ACT (e.g., multi-professional, available 24×7). Both ACT and CONG applied practices of harm reduction to risky behaviors (e.g., non-abstinence based substance use treatment). The model of service in CONG emphasized the promotion of community among residents, through activities such as on-site recreation (street hockey, basketball, yoga), preparation and sharing of meals, and organization of vocational opportunities (e.g., a graffiti removal business and positions associated with running the CONG building such as laundry, cleaning, and meal preparation). In addition, the CONG setting included the installation of a medical examination room and an on-site pharmacy, and was located near the city’s downtown entertainment district.

Treatment as usual (TAU) consisted of the existing and generally available services and supports for individuals experiencing homelessness and mental illness. During the course of the study, these resources included emergency shelters, housing units with varying levels of support, and various health and social service providers.

The study design included interviews every 3 months for 24 months. As part of the informed consent procedure, participants were assured that both HF interventions (SS and CONG) would be provided throughout the study period (i.e., at least 24 months of continuous housing and support). Participants were also advised that the study results would be used to advocate for reforms to housing and support services on an ongoing basis. Additional data were gathered from agencies responsible for publicly administered health, justice, and social services to citizens in British Columbia.

### Variables of Interest

The following socio-demographic variables collected at baseline are incorporated in the present analyses: gender; age; ethnicity (Aboriginal, White, Other); education; lifetime duration of homelessness; age first homeless; MCAS score; mental disorder status (type, severity, number of diagnoses); chronic health conditions; substance use disorder and infectious disease status. Our “severe” mental disorder cluster includes at least one of current (i.e. past month) psychosis, mood disorder with psychotic features, and hypomanic or manic episode, as identified through the MINI. The “less severe” cluster includes at least one of current major depressive episode, panic disorder, and post- traumatic stress disorder (PTSD). Substance dependence was also identified using the MINI. Infectious disease status was assessed based on a positive self-report diagnosis of HIV, Hepatitis B, or Hepatitis C.

Criminal convictions were drawn from administrative data representing all convictions extending from at least ten years prior to recruitment and up to two years post-randomization. Data include convictions in any Provincial court in the Province of British Columbia, and specify the type of offence. Offences were grouped as follows: property crimes; alcohol and drug related offences; and violent crimes. Follow up time was calculated from the difference between the randomization date and the study end date (March 31, 2012), and varied from a minimum of 9 months to a maximum of 24 months. The primary outcome of interest in this study was the number of convictions during the follow up period.

### Statistical Analysis

In order to evaluate the effect of HF interventions, an intention-to-treat analysis was conducted. Continuous variables (e.g., age, duration of homelessness) were reported in terms of means and standard deviations while categorical variables (e.g., gender, education level) were reported as proportions. Comparisons of variables between groups were conducted using independent sample t tests (continuous variables) and Pearson’s chi-square (nominal variables) tests where appropriate. Negative Binomial Regression (NBR) analysis was performed to model the independent association between the outcome variable (number of offences after randomization) and the primary independent variable (CONG, SS, TAU). NBR was chosen for the following reasons: count nature of outcome data, over-dispersion (higher variance compared to mean), and better goodness of fit statistics (compared to Poisson regression). Due to unequal length of follow up, log (natural) transformed follow up time was used as an offset variable in the regression analysis. Nested models and dispersion parameters were compared using the Likelihood Ratio (LR) test. Incidence Rate Ratios (IRR) obtained from the regression analysis were estimated per person. IRR (per person) along with 95% confidence intervals (CI) were reported as a measure of association. All reported p-values are two-sided.

The effects of HF interventions on recidivism were evaluated in univariate and multivariate settings. Variables that were controlled in multivariable models were chosen based on past research and a priori hypothesized relationships between the randomization conditions and recidivism. Covariates included in the multivariable model were housing interventions (CONG, SS & TAU); age at enrolment (continuous); lifetime duration of homelessness (continuous); age of first homelessness (continuous); number of offences in pre-enrolment (past five years) period (continuous); gender (male, female); ethnicity (Aboriginal, White, Other); high school education (yes, no); substance dependence (yes, no); less severe mental disorder (yes, no) and severe mental disorder (yes, no). Missing values for covariates that range from 0 to 2% were not included in the analysis.

IBM SPSS Statistics (Release Version 19.0, August 2010) and STATA 12 [32] were used to conduct these analyses.

## Results

In total, 297 individuals were recruited from October 2009 to June 2011. Within the total sample, 66% (n = 198) consented to the use of administrative data and had at least one recorded prior contact in the Provincial justice system, and were therefore eligible for inclusion in our analyses. Table 1 presents the results of baseline questionnaires for the full study cohort (n = 297) and also for the eligible participants (n = 198). The allocation of treatment status for the entire sample was as follows: CONG: 107; SS: 90; TAU: 100.

**Table 1:** Socio-demographic, mental disorder and physical illness related characteristics among participants.

Variable	All (n = 297) n (%)	Eligible (n = 198) n (%)
Study Arms		
Congregate (CONG)	107 (36)	78 (39)
Scattered Site (SS)	90 (30)	54 (27)
Treatment as Usual (TAU)	100 (34)	66 (33)
Male gender	218 (74)	150 (76)
Age at enrolment (in years) Mean (SD)	39.7 (11.2)	39.2 (10.3)
Ethnicity		
Aboriginal	44 (15)	36 (18)
White	170 (57)	104 (53)
Other	83 (28)	58 (29)
Education (less than high school)	179 (61)	125 (64)
Lifetime duration of homelessness (in months)		
Mean (SD)	62.0 (70.3)	63.0 (68.6)
Age of first homelessness Mean (SD)	28.7 (12.5)	27.7 (11.8)
MCAS score Mean (SD)	50.6 (6.7)	50.3 (7.0)
Multiple mental disorders (≥2)	148 (50)	104 (53)
Less severe cluster of mental disorder	133 (45)	94 (47)
Severe cluster of mental disorder	272 (92)	175 (88)
Substance dependence	183 (62)	128 (65)
Multiple physical illness (≥2)	231 (78)	157 (79)
Blood-borne infectious diseases (HIV/HCV/HBV)	87 (30)	68 (35)

The total sample was predominantly male (74%), with a mean age of 40 years, and self-identified ethnically as White (57%). Most participants reported not completing high school (61%), and experiencing over five years of homelessness in their lifetimes to date (mean = 62 months). Ninety-two percent of participants met criteria for mental disorders that were coded as “severe” (comprised of psychotic disorders and bipolar disorder), with nearly two-thirds (62%) also having substance dependence, and half (50%) having multiple mental disorders (not including substance dependence). Chronic health conditions were common among participants (78%) and nearly one-third (30%) reported having an infectious disease. The eligible or study sample (participants with prior justice system contacts, n = 198) did not differ significantly from the total sample on any of these variables (see Table 1).

The study participants (n = 198) committed an average of more than 8 offences in the 10 years before randomization (see Table 2). Half of these were property offences (4.09), followed by breaches of judicial orders (1.83). Drug and alcohol related offences accounted for a small proportion of the total number of convictions in the past 10 years (0.34). Table 2 also lists the numbers and types of offences in the 5 years, 2 years, and year prior to randomization. These results indicate a relatively stable rate of offending over multiple years within the sample.

**Table 2:** Offence-related characteristics among participants.

Variable	Eligible (n = 198)
Number of offences (any) before randomization	Mean (SD)
Last 10 years	8.07 (13.90)
Last 5 years	4.70 (7.19)
Last 2 years	2.17 (3.35)
Last year	1.25 (2.20)
Number of drug and alcohol related offences before randomization	Mean (SD)
Last 10 years	0.34 (1.09)
Last 5 years	0.20 (0.65)
Last 2 years	0.08 (0.35)
Last year	0.03 (0.20)
Number of breach offences before randomization	Mean (SD)
Last 10 years	1.83 (3.21)
Last 5 years	1.18 (2.31)
Last 2 years	0.56 (1.32)
Last year	0.33 (0.96)
Number of property offences before randomization	Mean (SD)
Last 10 years	4.09 (10.47)
Last 5 years	2.22 (4.86)
Last 2 years	1.02 (2.10)
Last year	0.59 (1.38)

A series of comparisons addressed the distribution of relevant variables between CONG, SS, and TAU. Results are presented in Table 3 and indicate that in each instance there were no significant differences between intervention arms at baseline.

**Table 3:** Comparisons of socio-demographic, mental disorder, physical illness and offence related characteristics by study arms among participants (n = 198).

Variable	CONG (78)	SS (54)	TAU (66)	P value
	n (%)	n (%)	n (%)	
Age at enrolment (in years)				
Mean (SD)	39.8 (10.6)	39.2 (10.3)	35.6 (10.1)	0.761
Lifetime duration of homelessness				
(in months) Mean (SD)	60.8 (68.2)	56.6 (57.8)	70.8 (76.9)	0.508
Age of first homelessness Mean (SD)	28.7 (11.7)	28.6 (12.0)	25.6 (11.6)	0.223
MCAS score Mean (SD)	49.6 (6.7)	51.7 (6.8)	50.2 (7.4)	0.235
Number of offences (any) before				
randomization (last 5 years)				
Mean (SD)	4.9 (7.3)	3.9 (5.9)	5.1 (8.0)	0.639
Number of offences (any) before				
randomization (last 2 years)				
Mean (SD)	2.3 (3.3)	1.4 (2.2)	2.7 (4.1)	0.119
Male gender	62 (80)	39 (74)	49 (77)	0.731
Ethnicity				
Aboriginal	18 (23)	8 (15)	10 (15)	
White	36 (46)	32 (59)	36 (55)	0.540
Other	24 (31)	14 (26)	20 (30)	
Education (less than high school)	52 (68)	28 (52)	45 (68)	0.115
Multiple mental disorders (≥2)	39 (50)	29 (54)	36 (55)	0.845
Less severe cluster of mental disorder	35 (45)	25 (46)	34 (52)	0.714
Severe cluster of mental disorder	70 (90)	47 (87)	58 (88)	0.881
Substance dependence	51 (65)	36 (67)	41 (62)	0.861
Multiple physical illness (≥2)	59 (76)	44 (82)	54 (82)	0.592
Blood-borne infectious diseases (HIV/HCV/HBV)	26 (34)	18 (33)	24 (37)	0.864

Offence rates following randomization were compared between study arms and in relation to selected additional variables. The unadjusted and adjusted IRR and 95% confidence intervals (CI) for each comparison are presented in Table 4. In comparison to TAU (reference), SS was associated with a significantly lower rate of convicted offences in the post period (Adjusted IRR = 0.29; 95% CI: 0.12–0.72). CONG was associated with a marginally significant lower rate of convicted offences than TAU (Adjusted IRR = 0.55; 95% CI: 0.26–1.14). The rate of convicted offences in the post period was also significantly associated with the rate of convicted offences in the 5 years prior to randomization (Adjusted IRR = 1.18; 95% CI: 1.11–1.26). In the adjusted model, several variables exhibited no significant relationship with recidivism following randomization, including: gender; ethnicity; educational achievement; age at enrolment; age first homeless; lifetime duration of homelessness; being diagnosed with substance dependence; being diagnosed with either a less severe mental disorder or a severe mental disorder.

**Table 4:** Negative Binomial Regression Analysis to estimate the Incidence Rate Ratio (IRR) of offence during the post-randomization period for the intervention arms among participants (n = 198).

Variable	Unadjusted IRR (95% CI)^1^	Adjusted IRR (95% CI)^2^
Study Arms		
Congregate (CONG)	0.58 (0.26, 1.33)	*0.55 (0.26, 1.14)* 3
Scattered Site (SS)	**0.23 (0.09, 0.60)**	**0.29 (0.12, 0.72)**
Treatment as Usual (TAU)	Reference	Reference
Age at enrolment (per year)	**0.96 (0.93, 1.00)**	0.98 (0.93, 1.02)
Male gender	**2.19 (1.02, 4.71)**	0.97 (0.42, 2.21)
Ethnicity		
Aboriginal	1.89 (0.74, 4.80)	0.45 (0.15, 1.35)
White	1.20 (0.57, 2.50)	0.95 (0.45, 2.00)
Other	Reference	Reference
Education (less than high school)	*1.83 (1.00, 3.47)* 4	0.79 (038, 1.67)
Age of first homelessness (per year)	**0.98 (0.95, 1.00)**	1.00 (0.95, 1.05)
Lifetime duration of homelessness		
(per month)	1.00 (1.00, 1.00)	1.00 (1.00, 1.01)
Number of offences (any) before		
randomization (last 5 years)	**1.17 (1.11, 1.23)**	**1.18 (1.11, 1.26)**
Substance dependence (yes vs. no)	*1.74 (0.91, 3.33)* 5	0.80 (0.39 1.63)
Less severe cluster of mental disorders	0.79 (0.42, 1.48)	0.73 (0.36, 1.47)
Severe cluster of mental disorders	0.92 (0.46, 1.84)	1.22 (0.42, 3.55)

1 Bold indicates significant at p≤0.05.

2 Bold indicates significant at p≤0.01.

3 Marginally significant (p = 0.108).

4 Marginally significant (p = 0.063).

5 Marginally significant (p = 0.095).

## Discussion

Our findings confirm that HF programs – particularly those using the scattered site format - promote reductions in offending and reconviction among people who were previously homeless and have a current mental disorder. Participants had been homeless for over five years and the vast majority (92%) experienced a psychotic disorder or (hypo)manic episode, reflecting the primary eligibility criteria for HF programs. An important critique of HF has centered on the under-representation of people with addictions in study samples, with a call to increase the inclusion of people with substance-related problems in research studies [33]. Two-thirds of our sample met criteria for substance dependence in addition to another mental disorder. However, the presence of a substance use disorder did not predict convictions post-randomization, indicating that non-abstinence based HF for people with concurrent disorders can effectively improve public safety. Consistent with the correctional literature, our adjusted model indicated that participants’ offending history was predictive of convictions following randomization [34].

Compared to usual care, participants in congregate and scattered site accommodations had, on average, 0.55 and 0.29 the number of reconvictions respectively following randomization. The effectiveness of both interventions may be attributable to potential positive impacts on myriad dynamic criminogenic factors when compared to usual care, including: direct exposure to crime; victimization; untreated mental disorders; poor food security and lack of opportunities for legal employment [35].

Compared to congregate housing, the lower reconviction rate among those in scattered site accommodations may be associated with differing neighborhood norms, undetected differences in support services, or differences in police practices and the probability of crime detection. Both interventions were sited in neighborhoods with diverse socio-economic and ethnic populations. Nevertheless, randomization to scattered sites entailed joining an established community with a mix of homes comprised of families, couples, and single tenants, and with high public order and low crime. By contrast, those participants randomized to the congregate setting became members of a new community, whose members all shared an immediate history of homelessness and mental illness, and with the full knowledge of neighborhood groups, police, and businesses. The CONG building was also situated in a more high-density downtown neighborhood proximal to the city’s “Entertainment District”, which does have a higher police-patrol presence relative to other neighborhoods. This may have resulted in a higher probability of detecting criminal offences in this group relative to those in the scattered site HF study arm. Though necessary for our research, the fact that the CONG building went from zero to full occupancy in a short period of time deviates from the regular operation of congregate housing, where participants are added periodically based on vacancies and the needs of those referred [36], and where membership in a stable, broader community can become established over time.

Neither the severity nor the number of mental disorders experienced by participants was associated with offending post randomization. Both HF interventions were therefore able to reduce reoffending regardless of a participant’s diagnostic status. This finding underscores the importance of addressing criminogenic risks that are shared by people who are homeless and mentally ill, such as poverty and exposure to crime, rather than triaging offence risk on the basis of specific symptoms [8]. Our results are consistent with broader social policy programs that have experimentally demonstrated improvements in physical and mental health among the poor through reductions in economic segregation [37].

Our findings underscore the consequences of failing to provide adequate housing and supports to homeless people with mental disorders. Over the ten years prior to recruitment, offenders in our sample were convicted roughly once per year, and typically for property offences. The relatively brief sentences elicited by these offences effectively disrupt individuals’ precarious accommodations, healthcare relationships and personal safety, thereby undermining recovery and perpetuating a proverbial “revolving door” [38]–[40]. Given the frequent contact of homeless mentally ill individuals with the courts, the objectives of both public safety and public health would be well served by establishing direct referral pathways for people who are homeless and mentally ill from the justice system to HF.

The study is limited by potential biases in self-reporting based on inaccuracies in recall or demand characteristics. Sources of data regarding reconvictions were restricted to the Province of British Columbia, and therefore do not reflect offences committed in other jurisdictions or those tried in Federal court. Although the services provided in both the SS and CONG settings were evaluated for consistency, it is possible that differences in services arose and are unaccounted for in the present analysis. Finally, it is possible that, in the post-randomization period, some participants committed offences but were not charged and/or were charged with offences but had not been convicted; however, this limitation would apply equally to individuals in each study arm.

This is the first randomized controlled trial to examine the longitudinal effect of HF on criminal convictions. Further, it is the first experiment to contrast congregate and scattered site versions of HF alongside usual care. And it is the first study to examine the association between HF and crime among chronically homeless mentally ill individuals with complex health and social needs, who comprise the core constituency served by HF programs in large urban centers. Inclusion criteria were satisfied through semi-structured interviews bolstered by collateral informants (e.g., diagnostic status from physicians), and dependent measures were obtained from the government agency responsible for all Provincial convictions in British Columbia.

## Conclusions

The results of our experiment demonstrate that HF produces significant reductions in reconvictions compared to usual care. People who are both homeless and mentally ill are frequently in contact with the justice system, a process that is both destabilizing to the individual and costly to society. The advent of HF has been shown to improve housing stability and health service involvement [5], [6], [41]–[44]. Our results extend the benefits of HF by showing improvements in public safety and reductions in crime.
